# K294E change in the rotavirus factory forming protein NSP2 stabilizes a rare C-terminal conformation

**DOI:** 10.1080/07391102.2025.2563689

**Published:** 2025-09-26

**Authors:** Sarah L. Nichols, Thomas Hollis, Freddie R. Salsbury, Sarah M. Esstman

**Affiliations:** a Department of Biology, Wake Forest University, Winston-Salem, NC, USA; b Department of Biochemistry, Wake Forest University School of Medicine, Winston-Salem, NC, USA; c Department of Physics, Wake Forest University, Winston-Salem, NC, USA

**Keywords:** Rotavirus, viroplasm, nonstructural protein, NSP2, molecular dynamics, X-ray crystallography

## Abstract

Rotaviruses (RVs) induce the formation of cytoplasmic viral factories, termed viroplasms, which are the sites of early particle assembly and viral RNA synthesis. The RV octameric nonstructural protein 2 (NSP2) plays critical, albeit incompletely understood, roles during viroplasm biogenesis. Previous work by our lab demonstrated that a RV bearing a lysine-to-glutamic acid (K294E) change in the flexible C-terminus of NSP2 exhibits defects in viral replication and induces smaller, more numerous viroplasms as compared to the wildtype (WT) virus. In this study, we sought to better understand if/how this K294E amino acid change altered the structure and/or dynamics of the NSP2 protein. We first determined the X-ray crystal structures of untagged, recombinant NSP2_K294E_ and NSP2_WT_. We found that both proteins formed highly similar octamers and crystallized in the I422 space group. To better understand the possible impacts of the K294E change on the conformations and backbone flexibility of NSP2, we performed molecular dynamics simulations. The results showed that NSP2_K294E_ adopted distinct C-terminal conformations relative to NSP2_WT_ and had subtle flexibility differences. Most notably, the data suggest that the K294E change stabilized a rare C-terminal conformation that was only infrequently sampled by NSP2_WT_. This shift in conformational preference may help explain why NSP2_K294E_ displayed decreased capacity to mediate robust viroplasm formation during RV infection. These results provide mechanistic insights into how a single amino acid change in the NSP2 C-terminus can have large effects on structural ensemble, shedding light on features of the protein that underpin RV viroplasm formation.

## Introduction

Rotaviruses (RVs) are important human pathogens and leading causes of acute gastroenteritis in children <5 years of age (Troeger et al., 2018). RVs are 11-segmented, double-stranded (ds) RNA viruses belonging to the *Sedoreoviridae* family (Matthijnssens et al., 2022). The RV virion is a nonenveloped, triple-layered particle comprised of 6 structural proteins (VP1–VP4, VP6, and VP7) (Estes & Kapikian, 2007; Settembre et al., 2011; Trask et al., 2012). An additional 5–6 nonstructural proteins (NSP1–NSP5 and NSP6 in some strains) are expressed during RV infection and have various roles (Estes & Kapikian, 2007). During host cell infection, RVs give rise to cytoplasmic viral factories, called viroplasms, which are the sites of early particle assembly and viral RNA synthesis (Papa et al., 2021; Patton et al., 2006; Taraporewala & Patton, 2004). Viroplasms have classic features of biomolecular condensates formed *via* the process of liquid-liquid phase separation (LLPS) (Geiger et al., 2021). NSP2 plays a critical role in the nucleation of viroplasms alongside its binding partner NSP5. NSP2 also has several activities within viroplasms, most notably functioning as an RNA chaperone to facilitate the assortment of the viral genome segments (Borodavka et al., 2017). Moreover, recombinant NSP2 exhibits *in vitro* nucleoside triphosphatase (NTPase) and RNA triphosphatase (RTPase) activities, as well as a nucleoside diphosphatase (NDPase) activity, which may be important for viral RNA synthesis (Kumar et al., 2007; Taraporewala et al., 1999; Vasquez-Del Carpio et al., 2006). Still, many of the conformational details that underlie NSP2’s various functions, including viroplasm formation, remain incompletely characterized.

**Figure 1.**
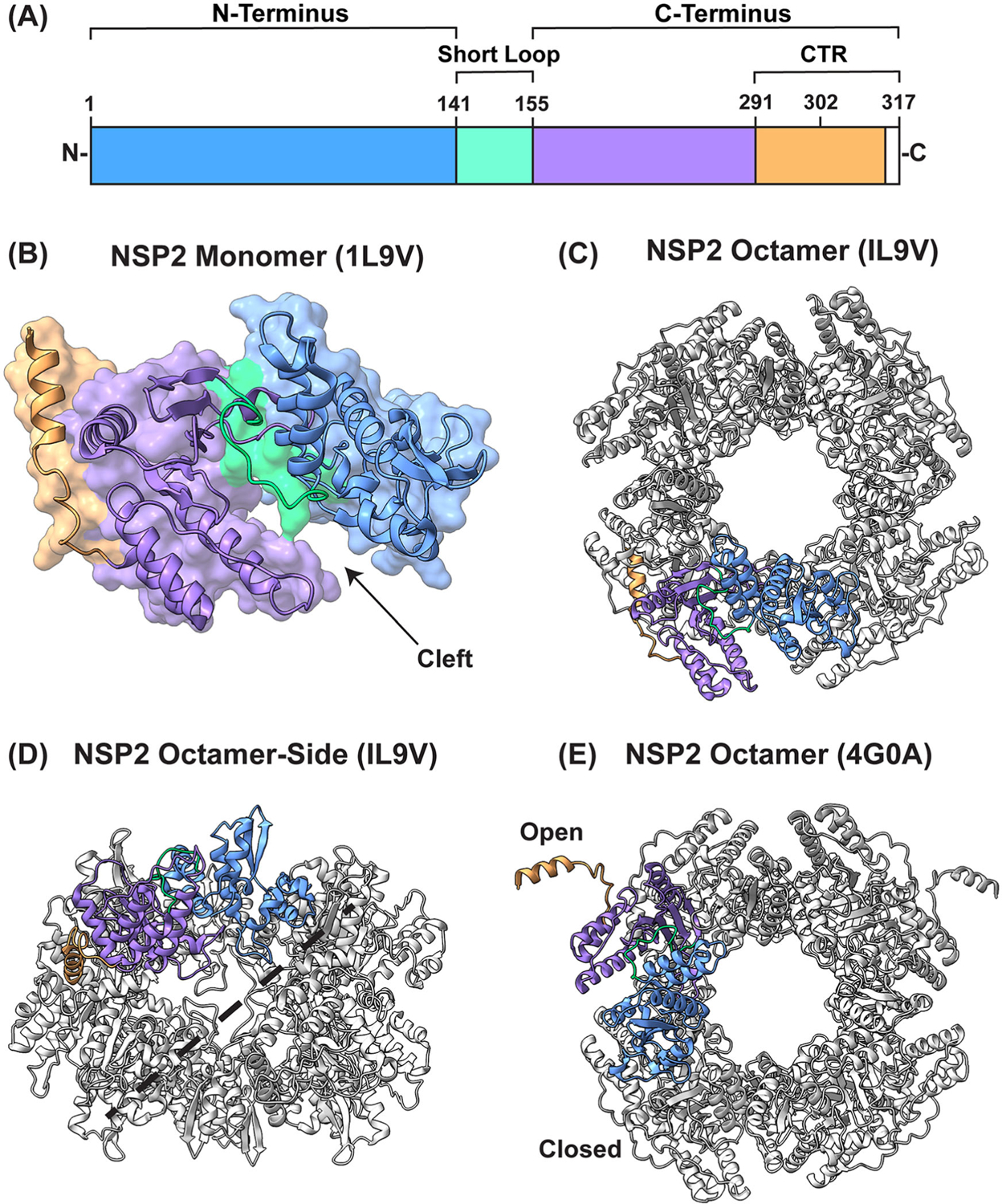
NSP2 structure. (A) Linear schematic of NSP2 (317 amino acids in length). The protein is comprised of two domains: an N-terminal domain (blue) and a C-terminal domain (purple and orange) separated by a short loop (turquoise). The extreme C-terminal region (res. ~291–317) is colored in orange. Residues 314–317 are unstructured (white). Adapted from Nichols et al. (2024). (B) NSP2 monomer (PDB no. 1L9V) as colored in (A) and is shown in both ribbon and surface representation. Catalytic cleft is indicated. Adapted from Nichols et al. (2024) and Jayaram et al. (2002). (C) NSP2 octamer structure (PDB no. 1L9V) shown in ribbon representation (light gray) with a monomer unit highlighted as colored in A and B. (D) NSP2 octamer from (C) is flipped backwards 90 degrees to show the side view along the two-fold axis. The electropositive groove where both RNA/NSP5 bind is represented by a dashed black line. (E) NSP2 octamer (PDB no. 4G0A) is shown in ribbon representation (light gray) with a monomer unit highlighted as colored in A and B. The open and closed positions of the CTR are indicated.

Several atomic structures of NSP2 have been determined using either X-ray crystallography or cryo-EM with single-particle reconstructions (Table 1). NSP2 is 35-kDa (~317 amino acids in length), and it forms a functional, donut-shaped octamer with a 35 Å-wide hole (Figure 1). Each NSP2 monomer unit has two distinct sub-domains that are connected by a short loop (residues ~141–155): (i) an N-terminal domain (residues ~1–140) and a (ii) C-terminal domain (~residues 156–313) (Figure 1(A,B)) (Jayaram et al., 2002). The N-terminal domain can be distinguished into a sub-domain consisting of two pairs of β-strands separated by two α-helices and another sub-domain consisting of four α-helices. These two N-terminal sub-domains are separated by a short, basic loop. The C-terminal domain adopts a twisted anti-parallel β-sheet followed by α-helices (Jayaram et al., 2002). The C-terminal region of NSP2 (CTR; residues 291–317) is comprised of a short linker region (residues 291–302) and alpha-helix (residues 303–313), with the extreme terminus being unstructured (residues 314–317) (Figure 1(A)). An essential feature of the NSP2 monomer is a 25 Å-deep cleft between the N- and C-terminal domains (Figure 1(B)). The catalytic residue H225, corresponding to the enzymatic activities of NSP2, exists within this cleft. One side of the cleft consists of a C-terminal α-helix and a flexible loop between residues ~247–266. The anti-parallel β-sheets of the C-terminal domain constitute the base of the cleft, along with an α-helix. The other side of the cleft consists of the inter-domain loop and an α-helix within the N-terminal domain. Two tetramers come together *via* tail-to-tail stacking interactions to form the functional octamer (Jayaram et al., 2002) (Figure 1(C,D)). As an octamer, these clefts coalesce as two highly basic, 25 Å-deep and 30 Å-wide grooves along each tetramer-tetramer interface. These electropositive grooves have been reported to be major binding sites for both RNA and NSP5 (Figure 1(D)) (Bravo et al., 2021; Jiang et al., 2006).

Most of the determined NSP2 structures exhibit D4 symmetry, whereby eight elements are related to each other by one 4-fold axis and two 2-fold axes. Moreover, nearly all X-ray crystal structures determined, both of C-terminally hexahistidine (cHis) tagged and untagged proteins, crystallize in I4 or I422 space groups (Table 1) (Bravo et al., 2021; Criglar et al., 2018; Jayaram et al., 2002; Kumar et al., 2007; Taraporewala et al., 2006). In these structures, the extreme CTR (residues 291–313) is in a closed position, oriented toward the body of the octamer, where it interacts with residues of the same monomer. However, an untagged NSP2 that was co-crystallized with an RNA dinucleotide deviated from this symmetry and crystallized into the P3_1_21 space group (PDB no. 4G0A) (Hu et al., 2012). In this structure by Hu et al., the octameric unit had the same general folding as all other structures; however, the extreme CTR of NSP2 existed in two different conformations (open and closed) (Figure 1(E)). In the open position, the CTR was shown to interact with a neighboring NSP2 octamer within the crystal lattice *via* domain-swapping interactions, thereby forming inter-octamer chains. The authors of this study speculated that CTR-mediated inter-octamer interactions were a key determinant for viroplasm formation. While the role of this novel CTR conformation remains to be experimentally validated, it is clear that this flexible region of the protein is important for viroplasm formation. More specifically, the deletion of nearly the entire CTR (residues 293–301) mitigated the formation of viroplasm-like structures in NSP2/NSP5 co-expressing cells (Criglar et al., 2014; Nichols et al., 2023). In addition to facilitating viroplasm formation, the flexible CTR has also been shown to play an important role in functional NSP2-RNA interactions (Bravo et al., 2021).

A previous study by our lab showed that a RV bearing a lysine-to-glutamic acid change at CTR position 294 (K294E) exhibited replication defects, and it induced significantly smaller and more numerous viroplasms as compared to the WT virus (Nichols et al., 2023). Moreover, the recombinant cHis-tagged NSP2_K294E_ protein showed a reduced capacity to mediate LLPS *in vitro* when incubated with recombinant NSP5 as compared to the control NSP2_WT_. However, this K294E amino acid change did not affect the NTPase activity of the recombinant protein *in vitro*. Thus, the K294E change in the CTR appeared to have impacted the capacity of NSP2 to form viroplasms, while leaving other functions of the protein intact. In this study, we sought to understand if/how the K294E change impacted NSP2 structurally or dynamically. We solved the X-ray crystal structures of untagged NSP2_K294E_ and NSP2_WT_, revealing that the overall folding of the proteins was highly similar (RMSD = 0.366 Å) and like those of other published NSP2 structures. Of note, both NSP2 proteins crystallized in the 1422 space group, meaning that the CTR was in the closed position for all monomers of the octamer unit. Thus, in contrast to the report by Hu et al., we did not detect evidence of inter-octamer interactions, even for untagged NSP2_WT_. We next turned to molecular dynamics (MD) simulations to shed light on possible conformational differences caused by the K294E change. This work revealed that the K294E change may have stabilized a rare, conformationally excited state of the NSP2 C-terminus, perhaps hindering the capacity of the protein to mediate interactions with its known binding partners (i.e. RNA and NSP5). These results provide new insights into structural and functional determinants within NSP2 that may be important for its function during viroplasm biogenesis.

## Results

### X-ray crystal structures of NSP2_WT_ and NSP2_K294E_ octamers

**Figure 2.**
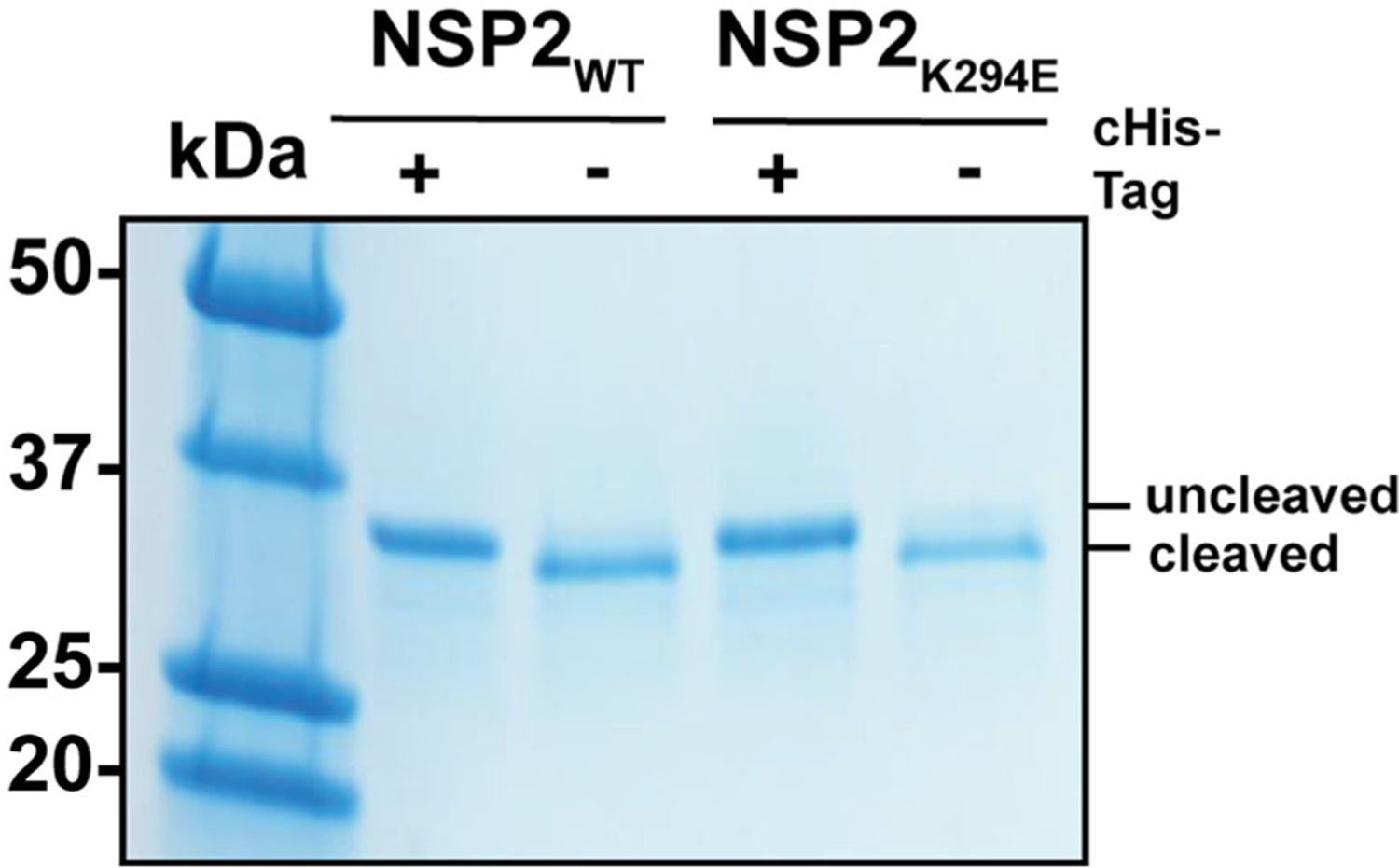
SDS-PAGE analysis of recombinant NSP2 proteins. Gel Code blue-stained Tris-glycine SDS-PAGE gel of nickel-column purified NSP2_WT_ and NSP2_K294E_ proteins either containing the cHis-tag (+) or with the cHis-tag removed (−) *via* thrombin cleavage. The molecular weight is indicated with Precision Plus All Blue Standard (BioRad).

The first structure of an NSP2_WT_ octamer (strain SA11) was determined with a cHis tag (PDB no. 1L9V) (Jayaram et al., 2002). A later study published by the same group revealed an X-ray crystal structure of SA11 NSP2 octamer without the cHis tag and in the presence of an RNA dinucleotide (GG), showing that the CTR of several monomers adopted a novel open conformation (PDB no. 4G0A) (Hu et al., 2012). To ensure that the conformation of the CTR in this study was not a result of the cHis tag, both NSP2_K294E_ and NSP2_WT_ control were crystallized as untagged proteins. To do this, the NSP2_K294E_ and NSP2_WT_ proteins were individually expressed in *E. coli* and purified using affinity chromatography. The cHis tags were then removed using thrombin cleavage upstream of the tag, and protein preparations were analyzed for cleavage efficiency and purity by SDS-PAGE (Figure 2). Following this, NSP2_K294E_ and NSP2_WT_ proteins were crystallized using previously published conditions (Jayaram et al., 2002). Structures were solved by molecular replacement using the previously determined NSP2_WT_ monomer structure (PDB no. 1L9V) as a search model, with the CTR removed to reduce bias. The data collection and refinement statistics for both NSP2 proteins are presented in Table 2.

**Figure 3.**
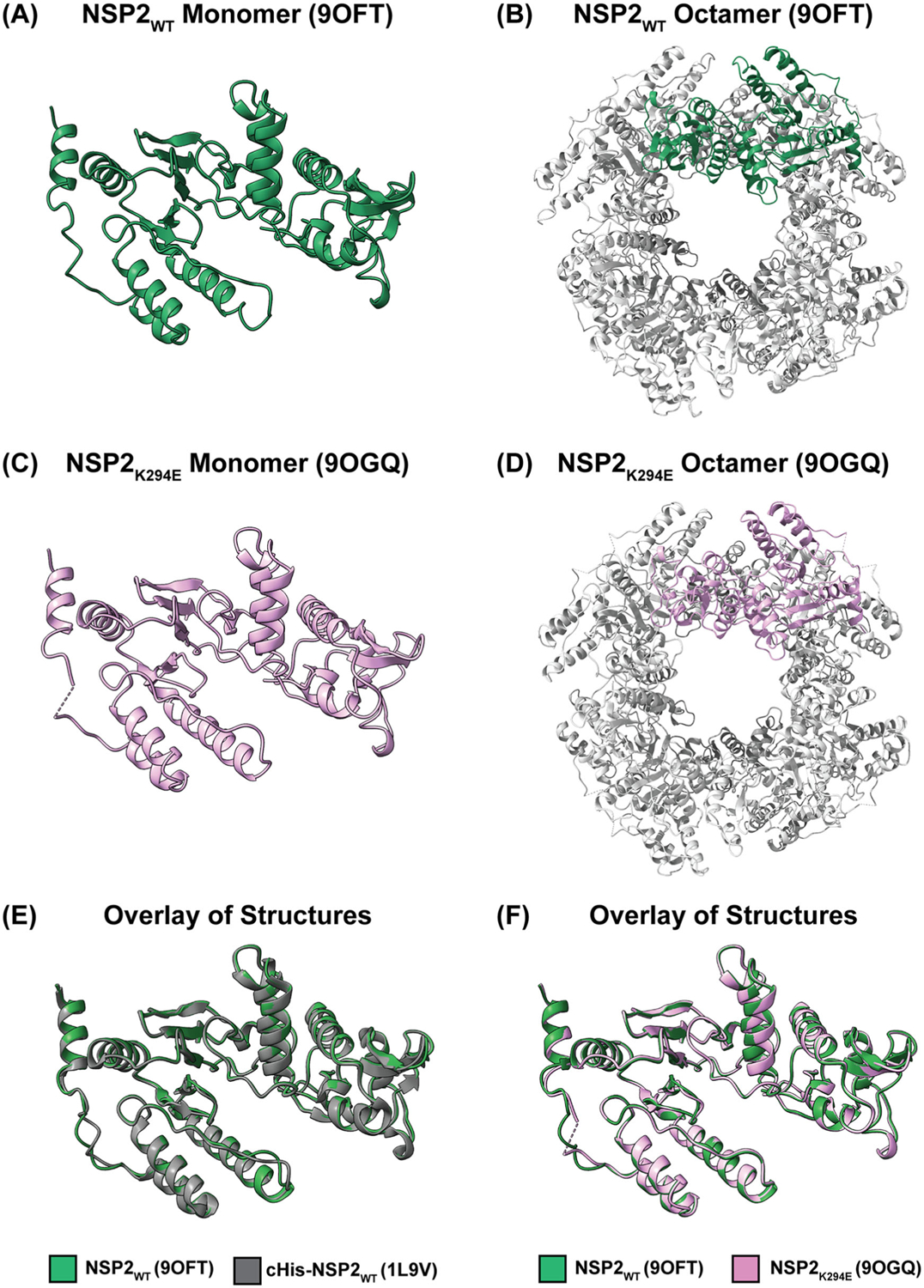
NSP2 proteins exhibit similar structures. (A) NSP2_WT_ monomer (PDB no. 9OFT) shown in ribbon representation. (B) NSP2_WT_ octamer (PDB no. 9OFT) shown in ribbon representation with a monomer unit highlighted in green. (C) NSP2_K294E_ monomer (PDB no. 9OGQ) shown in ribbon representation. (D) NSP2_K294E_ octamer (PDB no. 9OGQ) shown in ribbon representation with a monomer unit highlighted in pink. (E) Overlay of cHis-tagged NSP2_WT_ (PDB no. 1L9V; dark gray) and untagged NSP2_WT_ (PDB no. 9OFT; green) shown in ribbon representation. (F) Overlay of NSP2_WT_ (PDB no. 9OFT; green) and NSP2_K294E_ (PDB no. 9OGQ; pink) shown in ribbon representation.

The untagged NSP2_WT_ and NSP2_K294E_ protein structures remain consistent with previous structural studies of NSP2, as each protein crystallized into the I422 space group and readily formed octamers (Figure 3(A–D)). The NSP2_WT_ and NSP2_K294E_ octamers exhibit the traditional D4 symmetry (Figure 3(B,D)). Not unexpectedly, the structural features of the monomer units were highly conserved, and the superposition of the cHis-tagged NSP2_WT_ and untagged NSP2_WT_ depicts nearly identical folding patterns (RMSD = 0.332 Å) (Figure 3E). Moreover, the superposition of untagged NSP2_WT_ and NSP2_K294E_ suggest highly similar structures (RMSD = 0.366 Å) (Figure 3(F)). However, there is one notable structural difference; a portion of the CTR for NSP2_K294E_ is unstructured (residues 296–298) (Table 1; Figure 3(C,F)). Interestingly, all CTRs for NSP2_WT_ and NSP2_K294E_ crystallized in the closed position. Thus, in our hands, we did not see differences in the conformation of the CTR, suggesting that neither the cHis tag nor the K294E change affected the open v. closed CTR conformation, at least in the crystal structure. While we did not detect evidence of an open CTR position and NSP2 inter-octamer interactions in this study, there are differences between this work and the Hu et al. study that may account for this. Indeed, as aforementioned, in the Hu et al. study, NSP2 was co-crystallized with an RNA dinucleotide, and therefore, the resulting CTR conformations may be due to RNA binding.

### Regional flexibility differences in NSP2_K294E_

**Figure 4.**
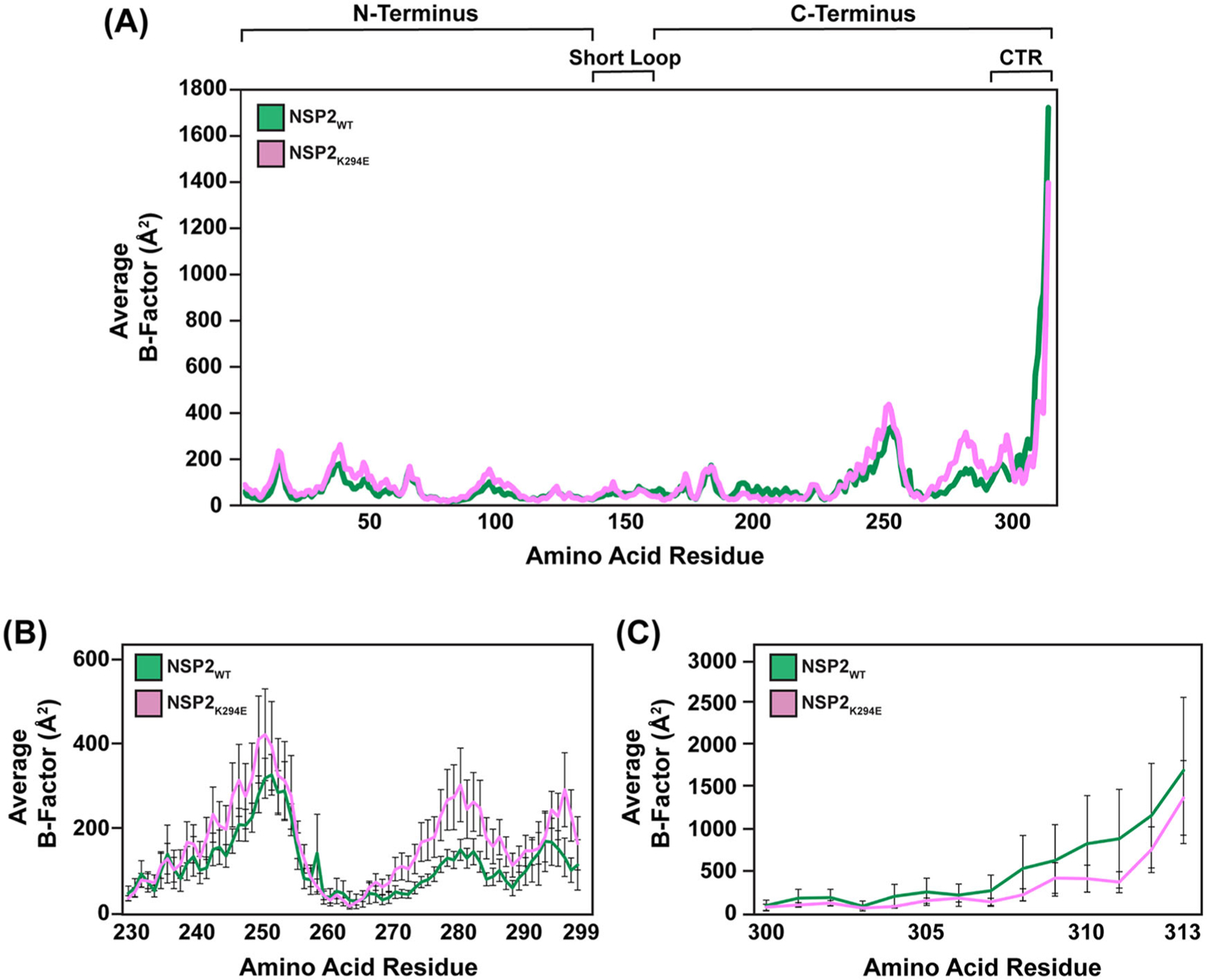
Subtle differences in flexibility of C-terminus between NSP2 proteins. (A) Line graph depicting the average B-factors across the entirety of the NSP2_WT_ (green) and NSP2_K294E_ (pink) proteins, where the Y-axis represents the average B-factor and the X-axis represents each amino acid residue (1–313). (B) Line graph depicting the average B-factors across C-terminal residues 230–299. Coloring same as panel A. Error bars represent standard error from mean. (C) Line graph depicting the average B-factors across C-terminal residues 300–313. Coloring same as panel A. Error bars represent standard error from mean.

Having observed that the overall X-ray crystal structures of NSP2_WT_ and NSP2_K294E_ were highly similar, we next turned to *in silico* MD simulations to tease out any possible dynamic/conformational differences. In our previous work, we performed 30-ns MD simulations on the cHis-tagged NSP2_WT_ structure (PDB no. 1L9V) and a homology model containing a K294E change (Nichols et al., 2023). In the current study, we have improved our methods in three ways: (i) we used the atomic structure of NSP2_K294E_ rather than a homology model, (ii) the cHis tag was removed from both proteins, and (iii) simulations were run- ~ three times longer (100 ns). In particular, four individual 100-ns simulations were performed on our solved NSP2_K294E_ and NSP2_WT_ monomer structures. Unstructured NSP2_K294E_ residues 296–298 were modeled for simulations (see methods). The root mean square fluctuations (RMSFs) of the α-carbons were used to calculate an average B-factor for each residue. A larger B-factor indicates more flexibility at a given residue and is used as one readout for conformational changes. The results suggest that there are generally similar levels of flexibility across the NSP2_WT_ and NSP2_K294E_ proteins, with subtle differences in the C-terminus (Figure 4(A)). Specifically, for C-terminal residues ~240–299, the data trends towards an increase in flexibility of NSP2_K294E_ as compared to the NSP2_WT_ control (Figure 4(B)). Of note, the data trends towards a decreased flexibility for a portion of the extreme CTR of NSP2_K294E_ (residues 300–313), consistent with our previously reported results with the homology model (Figure 4(C)) (Nichols et al., 2023). As B-factors can predict conformational changes in the protein backbone, these differences could indicate dynamic changes between the two proteins.

### NSP2_K294E_ and NSP2_WT_ exhibit disparate conformational ensembles

**Figure 5.**
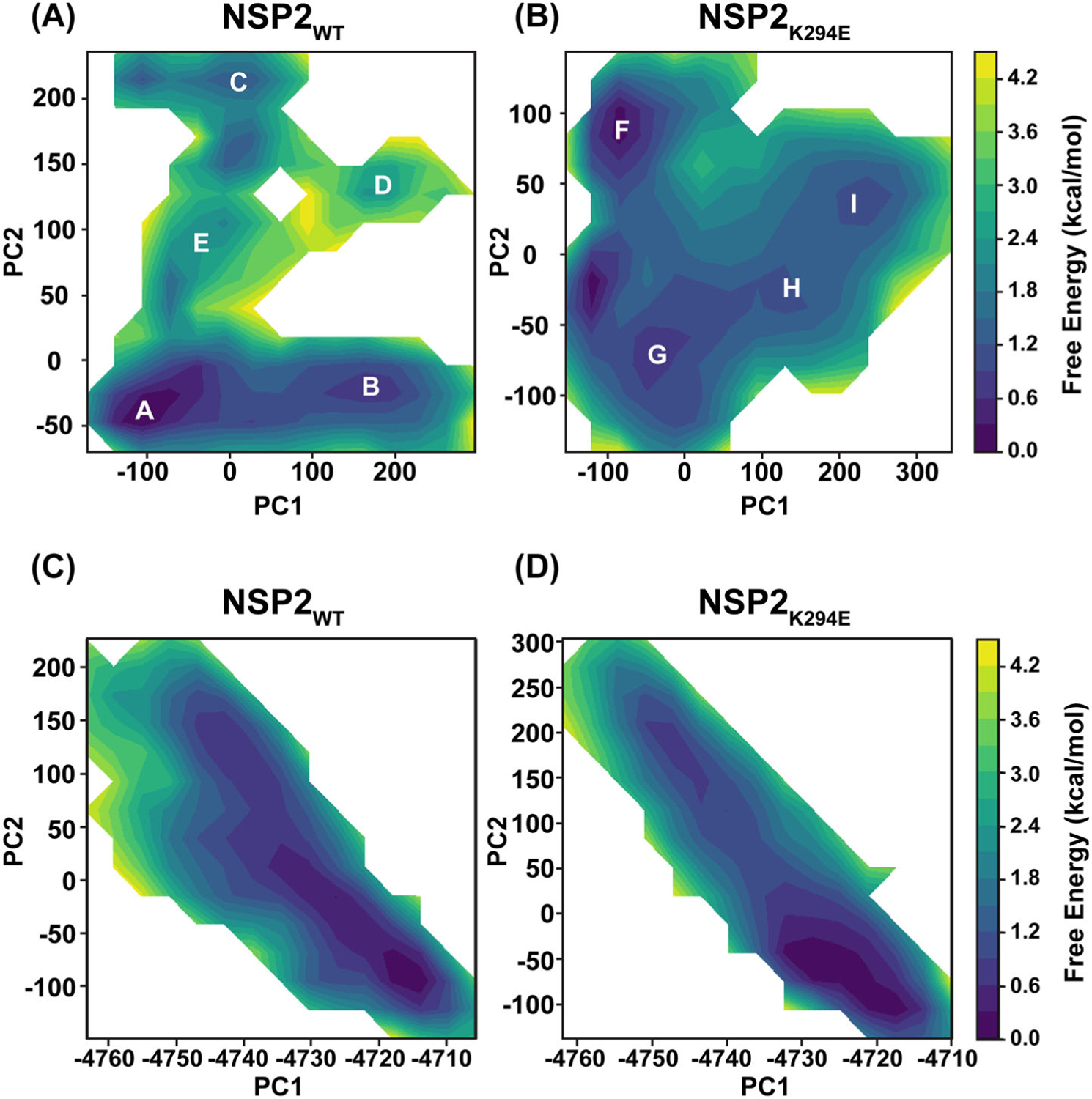
Differences in NSP2_WT_ vs. NSP2_K294E_ conformations. (A, B). The PCA plot for NSP2_WT_ shows that five conformations were taken during simulations, while the PCA plot for NSP2_K294E_ shows that four conformations were taken during simulations. For NSP2_WT_, A = the most stable conformation, followed by B, C, D, and E. For NSP2_K294E_, F = the most stable conformation, followed by G, H, and I. The associated free energy (kcal/mol) is represented by a simple color scale, whereby deep blue indicates lower free energy, and higher conformation stability, while light green/yellow represents higher free energy and lower conformation stability. (C, D). PCA of the common atoms of NSP2_WT_ and NSP2_K294E_, whereby the principal components on the common atoms were calculated, and each trajectory was separately projected onto the two lowest common principal coordinates.

**Figure 6.**
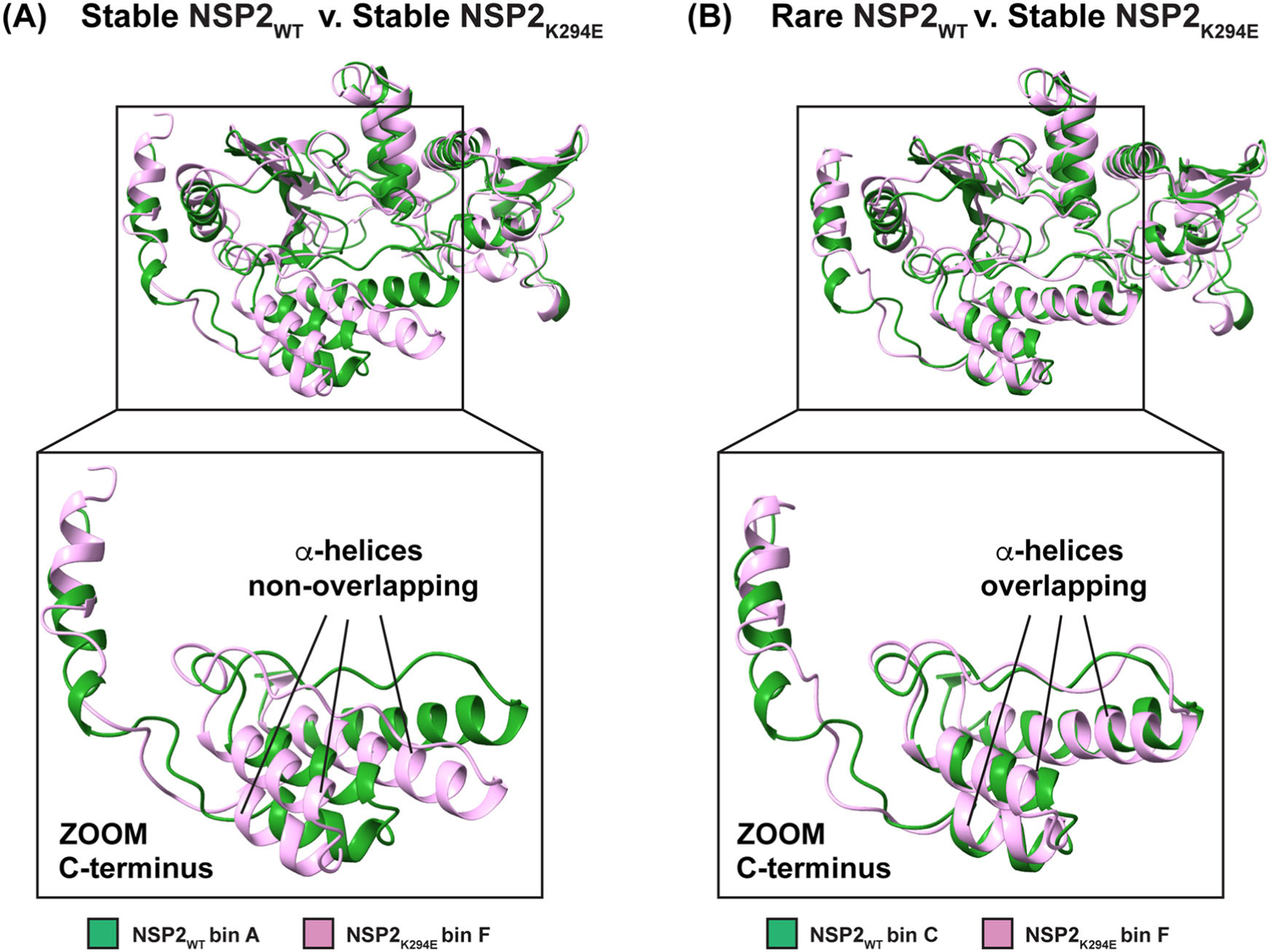
NSP2_K294E_ stabilized an excited state of the C-terminus. (A) Overlay of the most stable NSP2_WT_ conformation (bin A) and NSP2_K294E_ conformation (bin F). (B) Overlay of rare NSP2_WT_ conformation (bin C) and most stable NSP2_K294E_ conformation (bin F). A zoomed-in panel of the C-terminus is shown below each overlay, and the three α-helices showing the most changes are indicated.

To provide a deeper understanding of the possible impact(s) of the K294E change on the NSP2 structure, we next sought to determine the predominant conformations of NSP2_WT_ v. NSP2_K294E_ during molecular dynamics simulations. For ease of comparison, the conformational data were organized into principal component analysis (PCA) plots. The plots are labeled with letters (A-E for NSP2_WT_ and F-I for NSP2_K294E_), where each letter represents a ‘bin’ of highly similar conformations that the protein took throughout the simulation (Figure 5). The conformations found in each bin can be represented by the centroid structure and then compared to each other. Thus, we used this approach to make pairwise comparisons between these representative structures taken by NSP2_WT_ and NSP2_K294E_ throughout the simulations (Figure 6, Table 3, and supplemental data). The stability of each conformation can be evaluated by its free energy, which informs how often the conformation would be expected to occur in the population. Specifically, lower free energy indicates a higher stability and a higher frequency of a conformation. The free energy scale acts as a simple color guide to evaluate stability, where a deeper blue color indicates lower free energy and a more stable conformation. In contrast, an increase in free energy on this scale is represented by a light green or yellow color, indicating a less stable conformation or a more excited conformational state of the protein (Figure 5). Thus, for NSP2_WT_, bin A represented the most stable conformation, followed by bins B, C, D, and E (Figure 5(A)). Similarly, for NSP2_K294E_, bin F represented the most stable conformation, followed by bins G, H, and I (Figure 5(B)). We also performed a PC analysis with the common atoms of NSP2_WT_ and NSP2_K294E_ where we calculated the principal components on just the common atoms and projected each trajectory separately onto the two lowest common principal coordinates. Although details are lost compared to analyzing each separately, these plots (Figure 5(C,D)) do show that while there is a great deal of commonality, NSP2_K294E_ does stabilize regions that are not in a minima in the NSP2_WT_ ensemble. These results show that the two NSP2 proteins are predicted to have different conformational free energy surfaces, suggesting differences in the conformations they adopted during simulations. To evaluate this further, we performed a pairwise comparison of each dominant conformation for both proteins (Table 3 and supplemental data). Interestingly, the most stable, lowest energy conformations (bin A for NSP2_WT_ and bin F for NSP2_K294E_) had predicted structures that are very different from each other (RMSD = 2.663 Å). In fact, the most prominent differences between these structures map to the C-terminus (residues ~230–313), wherein a cluster of three α-helices show almost no overlap (Figure 6(A)). A pairwise comparison of the lowest energy, most stable conformation for NSP2_K294E_ (bin F) with other NSP2_WT_ conformations revealed that superposition with bin C has the lowest RMSD value (RMSD = 2.015 Å) (Table 3). Upon structural comparison of these two conformers, we determined that the NSP2_WT_ conformation of bin C was most similar to that seen with NSP2_K294E_ (bin F), with three α-helices in the C-terminal region closely overlapping (Figure 6(B)). Of note, this C-terminal conformation was rare in the NSP2_WT_ population and, therefore, represented a more excited state of the protein. To quantify this, we constructed multistate models for the two ensembles based on the free energy surfaces shown in Figure 5 by calculating Boltzmann weights using the free energies corresponding to the lowest-energy bin within each minimum. The resulting population distributions are summarized in Table 4. In both ensembles, two dominant structural states are observed with comparable populations, although additional, less-populated states are also present. Importantly, NSP2_WT_ bin C occurs ~15.7% of the time, corroborating the notion that this conformational state is rare for the wildtype protein, whereas it is the most common state for NSP2_K294E_ (Table 4). Altogether, these results suggest that the K294E change may have stabilized an excited, rare conformational state of the NSP2 C-terminus.

## Discussion

RV NSP2 is critical for the formation and function of viroplasms, which are the sites of early particle assembly and viral RNA synthesis inside the infected cell. These viral factories are membraneless biomolecular condensates nucleated by NSP2 and its cognate binding partner NSP5. While molecular determinants within NSP2 that are important for its function(s) remain incompletely understood, the NSP2 CTR (residues ~291–313) has been proposed as a crucial element for viroplasm formation (Bravo et al., 2021; Criglar et al., 2014; Nichols et al., 2023). The first published X-ray crystal structure of an NSP2 octamer was of the cHis-tagged protein, and it crystallized into the 1422 space group, whereby all monomer units within the octamer were identical (Figure 1(B)) (Jayaram et al., 2002). However, in a later study, this same group published the X-ray crystal structure of untagged NSP2, and in this case, the CTRs of various monomers adopted open and closed conformations (Figure 1(E)) (Hu et al., 2012). In the open conformation, the CTR interacted with a neighboring octamer of the crystal lattice, resulting in inter-octamer chains (Hu et al., 2012). The authors of this study postulated that this phenomenon could underpin viroplasm formation. Changes in the backbone angles of CTR residues M293, K294, and P295 are thought to underpin this switch between the open v. closed CTR states (Hu et al., 2012). In our previous study, we introduced a single K294E amino acid change, creating a RV with a small viroplasm phenotype and an NSP2 protein with an *in vitro* LLPS defect (Nichols et al., 2023). Given the report by Hu et al., we posited that the K294E change may have impacted the capacity of the CTR to adopt open v. closed positions, in turn affecting inter-octamer interactions, LLPS, and viroplasm formation. Therefore, in this study, we sought to determine the X-ray crystal structure of untagged NSP2_K294E_ v. untagged NSP2_WT_ with the goal of determining whether there are differences in the CTR positions between the two proteins.

Interestingly, our results with untagged NSP2_WT_ differed from those reported by Hu et al. (2012). Specifically, the NSP2_WT_ protein (as well as NSP2_K294E_) crystallized in the I422 space group, with the CTRs of all monomers in the closed position. This closed CTR state was identical to that of the cHis-tagged structure reported by Jayaram et al. (2002). Thus, we conclude that neither the cHis tag nor the K294E change affected the conformation of the CTR, at least in the context of X-ray crystallography. Of course, our new structural results raise two questions: Does the NSP2 CTR adopt this open state? Does NSP2 form octamer chains, or is this structural event a crystallographic packing effect? It is certainly possible that the open position of the CTR may require specific conditions not recapitulated in our study. In particular, in addition to removing the cHis tag, Hu et al. also co-crystallized NSP2 with an RNA dinucleotide (GG), which could have influenced the propensity for the CTR to adopt the open conformation. It is alternatively possible that the position of the CTR in the Hu et al. study was the result of the protein in an unusual crystalline state, which could have resulted in nonphysiological contacts with neighboring proteins in the crystal (Terada & Kidera, 2012). Future biochemical studies, such as those using sucrose gradient sedimentation or size-exclusion chromatography with multi-angle light scattering (SEC-MALS), will be critical to clarify whether the NSP2 CTR can adopt the open state and whether NSP2 forms inter-octamer chains in a more biologically relevant environment.

**Figure 7.**
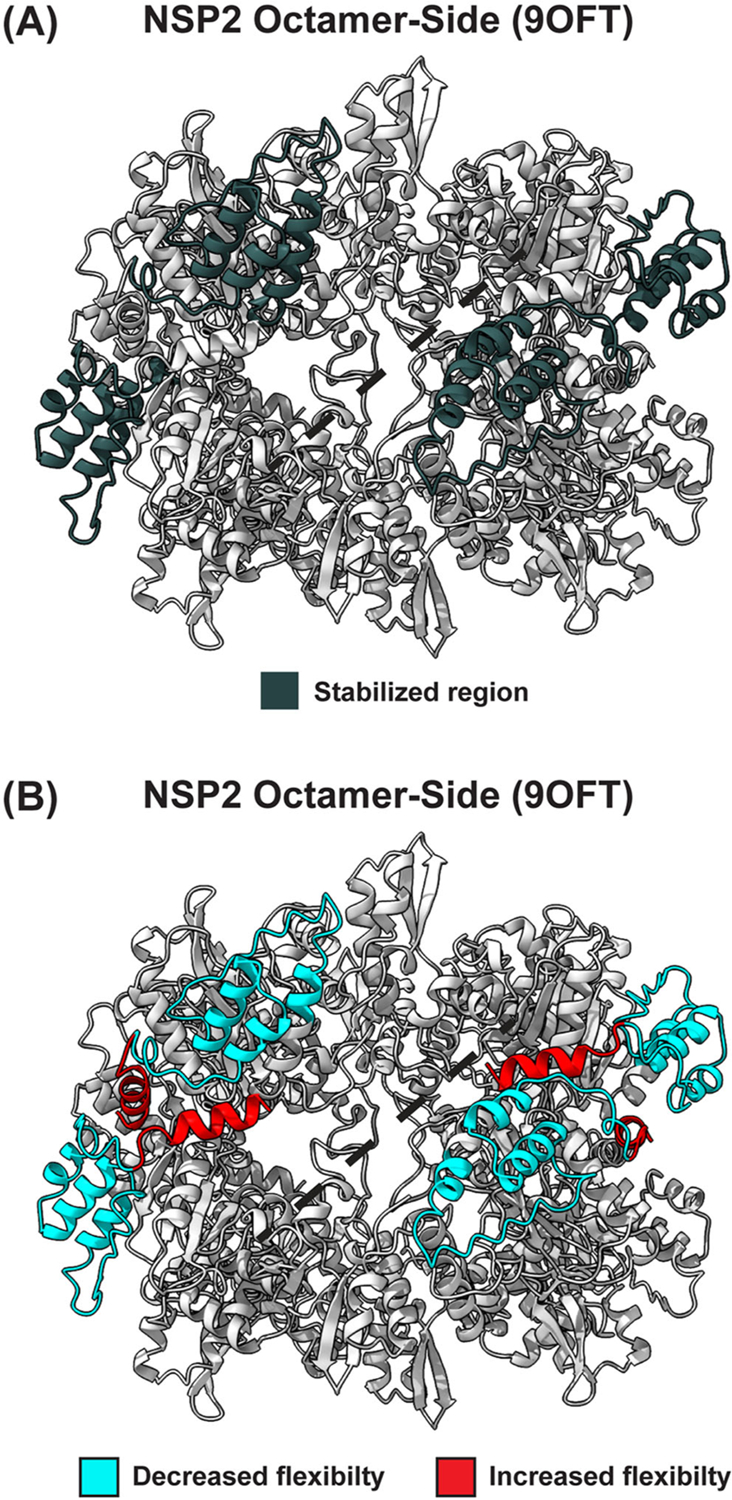
Visualization of the affected C-terminal regions. (A) NSP2 octamer (PDB no. 9OFT) is shown in ribbon representation in gray, with C-terminal residues 230–313 highlighted in black. The electropositive groove is represented by a black dashed line. (B) NSP2 octamer (PDB no. 9OFT) is shown in ribbon representation in gray, where C-terminal residues associated with increased flexibility are highlighted in red (residues ~240–299), and those associated with decreased flexibility are highlighted in blue (residues 300–313). The electropositive groove is represented by a black dashed line.

Because the crystal environment can influence the protein structure and create nonphysiological contacts, it is important to predict the solution structure of a protein using MD simulations (Terada & Kidera, 2012). Because the NSP2_WT_ and NSP2_K294E_ protein structures in our study appeared highly similar, we wondered whether MD simulations could predict any differences. The results support the notion that a K294E change affects the CTR, a known molecular determinant of viroplasm formation, and posits that additional residues within the C-terminus are important for NSP2 function. More specifically, our data predicted that the most common conformational state of NSP2_K294E_ is different from that of NSP2_WT_, and the region exhibiting the largest difference maps to the C-terminus (residues ~230–313) (Figure 7(A)). While a similar C-terminal conformation adopted most commonly by NSP2_K294E_ is predicted to exist in the NSP2_WT_ population, this conformation is rare, occurring ~15.7% of the time (Table 4). Indeed, in NSP2_WT_, this C-terminal conformation likely represents a more excited state of the protein, given that it is associated with a high free-energy value. Thus, based on this data, we now hypothesize that the K294E change may have stabilized an excited, rare conformation of the NSP2 C-terminus. The associated conformational changes align with the predicted changes in regional flexibility, whereby average B-factors trend towards an increase in flexibility in the C-terminus (residues ~240–299) and a decrease in flexibility in part of the extreme CTR (residues ~300–313) of NSP2_K294E_ as compared to NSP2_WT_ (Figure 7(B)). This trend towards decreased flexibility in the extreme CTR is similar to what was seen previously with a homology model (Nichols et al., 2023). At this time, we do not have a clear explanation for what is driving the structural dynamics changes. Similar hydrogen bond patterns were found within NSP2_WT_ versus NSP2_K294E_ when analyzing the static crystal structures and the dominant simulated conformations (data not shown). It is possible that the positive (K) to negative (E) amino acid change influenced the observed dynamic differences, and biochemical and biophysical experiments—such as hydrogen-exchange mass spectrometry or FRET distance measurements—that would test how the K294E charge reversal modulates NSP2’s internal interactions would be a logical future step.

The results of this current study also led us to speculate how a change in the conformational state and/or flexibility in the C-terminus impacted NSP2’s role in viroplasm biogenesis. The answer may relate to the regions of NSP2 known to bind NSP5 and RNA, in particular the electropositive groove (Bravo et al., 2021; Jiang et al., 2006; Nichols et al., 2023). It is known that NSP5 is crucial for viroplasm nucleation, and it is likely that RNA plays a role in this process as RNA is a known LLPS inducer (Alberti et al., 2019; Eichwald et al., 2004; Geiger et al., 2021; Papa et al., 2021). Moreover, both are known to interact with NSP2 directly; in fact, structural studies have identified a series of NSP2 residues that are predicted to interact with RNA (residues 43, 58–60, 68, 184, 230, 240, 242, 245, 286, 290, and 300) and/or NSP5 (residues 64–68; 179–183; 232–251; and 291–313) (Bravo et al., 2021; Jiang et al., 2006). Interestingly, some of these NSP5- and RNA-binding residues fall within the region of the C-terminus that map to the induced conformational changes, very near the electropositive groove. Specifically, RNA binding residues 286, 290, and 300, as well as NSP5 binding residues 232–251 and 291–313, overlap with regions identified in this study. While disrupted RNA interactions may contribute somewhat to the K294E viroplasm phenotype, we think that altered NSP5 interactions are a major driver. In particular, in our previous study, we investigated NSP2’s ability to phase separate with NSP5 using an *in vitro* LLPS assay (Nichols et al., 2023). From these assays, we concluded that NSP2_K294E_ was impeded in its capacity to mediate efficient LLPS with NSP5. Our data gathered herein corroborate this notion, as our crystal structures suggest that NSP2 does not form inter-octamer chains, positing diminished NSP2-NSP5 interactions as the likely cause of the K294E viroplasm phenotype.

LLPS can be summarized as the product of weak, transient, multivalent interactions of molecules (Wang et al., 2021). These individually weak interactions collectively lower global free energy, which is thought to promote demixing and drive phase separation (Bentley et al., 2019; Berry et al., 2018; Krainer et al., 2021; Wang et al., 2021). Given the opposite charges of NSP2 and NSP5 and the likely LLPS-prone regions, hydrophobic and electrostatic (cation-anion and ionic bonds) forces are likely driving phase separation of these proteins (Geiger et al., 2021; Nichols et al., 2023). Significantly, our data predicts that the K294E change imparts global C-terminal conformational changes associated with changes in flexibility and a higher free-energy state. Given that these charge-based multivalent interactions and the associated free energy drive LLPS, it is interesting to hypothesize whether the charge change, predicted C-terminal conformational changes, and the increased free energy corresponding to the K294E change result in the decreased capacity of NSP2 to engage in multivalent interactions with NSP5. Future biochemical and biophysical experiments, or additional structural studies of NSP2 and/or NSP5 or RNA bound together, will be required to tease out these mechanisms. Nevertheless, the knowledge gained here sheds light on the impact of point mutations on viral protein structure and dynamics. Also, it highlights the limitations of structural biology and the nuance that computational tools can provide to understand native structural states. This work has moved us closer to understanding this complex process of viroplasm formation and provided us with regions and residues of NSP2 that may support this process.

## Materials and methods

### Large-scale purification of recombinant NSP2 proteins

The engineered pET-28a-NSP2_WT_ and pET-28a-NSP2_K294E_ vectors containing a strain SA11 NSP2 open reading frame followed by a C-terminal thrombin cleavage site upstream of a cHis tag were engineered previously (Nichols et al., 2023). The constructs were used to express cHis-tagged NSP2_WT_ and NSP2_K294E_ proteins, respectively, in *E. coli* Rosetta II cells. Bacteria were grown at 37 °C in a shaking incubator until an optical density at 600 nm (OD_600_) of 0.4–0.6 was reached, at which point isopropyl β-_D_-1-thiogalacto-pyranoside was added at a final concentration of 1 mM to induce NSP2 expression. Following a 4 h induction at 37 °C, bacteria were harvested *via* centrifugation at 6227×*g* for 30 min at 4 °C in the fixed-angle rotor JLA 8.1000. Pelleted bacteria were resuspended in lysis buffer (100 mM Tris-HCl, pH 7.5, 500 mM NaCl, 20 mM imidazole, 0.1% Triton X-100) containing EDTA-free protease inhibitor minitablets (Pierce). Next, bacteria were homogenized using a high-pressure homogenizer (15,000 psi for 10 min). Following homogenization, the lysate was clarified by centrifugation in the fixed-angle rotor JA-25.50 at 39,121×*g* for 30 min at 4 °C. The clarified lysate was run through a nickel resin column and washed with 1 L of wash buffer (100 mM Tris-HCl, pH 7.5, 500 mM NaCl, 20 mM imidazole). The NSP2 proteins were eluted with elution buffer (100 mM Tris-HCl, pH 7.5, 500 mM NaCl, 250 mM imidazole) and exchanged to storage buffer (20 mM Tris-HCl, pH 7.5, 0.5 mM EDTA). Proteins were frozen overnight in storage buffer supplemented with 20% glycerol at −80 °C and transferred to −20 °C until needed. His tag was removed using the Thrombin Cleavage Capture Kit (EMD Millipore) according to the manufacturer’s instructions. Proteins were concentrated to 20–30 mg/mL using Amicon Ultra Centrifugal Filters (Millipore Sigma) and analyzed by electrophoresis in 4–15% Tris-glycine SDS-PAGE gels (Bio Rad) stained with Gel Code blue according to the manufacturer’s instructions.

### Crystallization conditions

The purified NSP2_K294E_ and NSP2_WT_ proteins in 10 mM Tris-HCl, pH 7.2, 50 mM NaCl, and 1 mM DTT were each crystallized by the sitting drop vapor diffusion technique using a reservoir solution consisting of 100 mM Tris-HCl, pH 6.5–8.0, 0.2 M magnesium acetate, and 8–18% PEG6K. Specifically, ~8 mg/mL of each protein was equilibrated as a 1:1 mixture with the reservoir solution. In all cases, crystallization was carried out at 20 °C. Crystals appeared within 1–4 days, and data were collected ~3 wk post-plating. Prior to data collection, crystals were soaked in reservoir solution with 5%, 10%, and 20% glycerol for about 1 min each prior to cryo-cooling. The crystals were then mounted in a nylon loop and cryo-cooled in liquid nitrogen. The reservoir solution for diffracted crystals for both proteins was 100 mM Tris-HCl, pH 7.5, 0.2 M magnesium acetate, and 12% PEG6K. Crystals of the untagged NSP2_WT_ structure belong to space group I422 with unit cell dimensions a = *b* = 108.6, *c* = 153.4, α = β = γ = 90°. Crystals of the untagged NSP2_K294E_ structure belong to space group I422 with unit cell dimensions a = *b* = 108.4, *c* = 153.9, α = β = γ = 90°.

### Structure determination and refinement

X-ray data were collected using CuK radiation on a MicroMax 007 generator and a Pilatus 3 R detector (Dectris). Data reduction and integration were performed in the HKL3000 program. Phases for the data were obtained by molecular replacement using the program Phaser and a monomer of the SA11 NSP2 protein (PDB no. 1L9V) as a search model. All NSP2 structures were built using the program Coot and the structures were refined in Phenix. The change in the free R-factor was monitored at each step in refinement, as well as the inspection of stereochemical parameters with the programs Coot and Phenix. The models converged with a final R-factor of 23.8% for the untagged NSP2_WT_ (R_free_ = 26.9%) and an R-factor of 22.6% for the untagged NSP2_K294E_ (R_free_ = 25.2%) using all observed X-ray data measurements in the resolution range of 34.37–2.95 and 46.39–2.2 Å, respectively. Further data collection and refinement statistics are shown in Table 2. Coordinates were deposited for untagged NSP2_WT_ and untagged NSP2_K294E_ with accession codes 9OFT and 9OGQ, respectively.

### In silico *MD simulations*

All MD simulations were performed using ACEMD software (Harvey & De Fabritiis, 2009), and monomer units of the solved NSP2_WT_ or NSP2_K294E_ structures. Since the Leu-Val-Pro-Arg peptide that would remain after thrombin cleavage is absent in those structures, it was omitted from the simulations. Prior to performing simulations, structures were solvated with a three-point water model (TIP3P) (Jorgensen et al., 1983) in a cubic box with a minimum buffer of 10 Å in all directions, and charges were neutralized by chloride ions. The CHARMM36 forcefield (Huang & MacKerell Jr, 2013) was used for all simulations. The pressure of the system was maintained at 1 atm using the Berendsen barostat pressure control method (Berendsen et al., 1984; Harvey & De Fabritiis, 2009), and the temperature was maintained at 300 K using a Langevin thermostat approach, with a damping coefficient of 0.1 applied (Harvey & De Fabritiis, 2009). The computation of long-range electrostatics was performed using the smooth-particle-mesh-Ewald (PME) method (Darden et al., 1993; Harvey & De Fabritiis, 2009). The SHAKE algorithm, a widely accepted approach for constraining bond lengths to hydrogen atoms, was employed to ensure efficient integration (van Gunsteren & Berendsen, 1977). Starting structures were energy minimized for 1000 steps of conjugate gradient minimization. An unrestrained 100 ns NPT molecular dynamics simulation was run at 300K. We ran four 100 ns simulations for each system, each with different initial velocity seeds, collectively amassing 100,000 frames per system. To obtain B-factors, individual RMSFs were converted using the following formula (RMSF)^2^*((8*π^2^)/3). The B-factors were then averaged among individual residues, and the standard error of the mean was calculated. To determine significance, a two-tailed F-test was first performed to determine whether there were equal vs. unequal variances. Following this, a two-tailed, unequal variance t-test was performed. *p* < 0.05 were considered statistically significant.

Principal component analysis (PCA) was employed to characterize conformational changes in the system. By projecting the trajectory onto a small set of orthogonal principal components, PCA captures the dominant modes of structural variation and thus simplifies the interpretation of complex, high-dimensional data (Abdi & Williams, 2010).

### PCA procedure

All trajectories were first fitted to the initial crystal model (Ca atoms) to remove overall translation/rotation. For every frame, we then constructed the 3 N-dimensional coordinate vector $\text{r}=\left({\text{x}}_{1},{\text{y}}_{1},{\text{z}}_{1},\ldots ,\;\text{x}\_\text{N},\;\text{y}\_\text{N},\;\text{z}\_\text{N}\right)$ of the atoms. Following Wu and Salsbury (2022), the coordinates of T aligned frames were stacked into a T×3 N matrix A, centered, and the covariance matrix $\text{C}=\left({\text{A}}^{\text{T}}\;\text{A}\right)\text{/}\left(\text{T}-1\right)$ was diagonalized to obtain eigenvectors v_i (principal components, PCs) and eigenvalues λ_i. The first two PCs (PC1, PC2) capture 46% of the total variance of the wildtype simulation and 48% of the total variance of the K294E mutant—comparable but higher than the values reported by Wu and Salsbury (2022) in a similar analysis on a different protein. For the joint PC analysis, the common atoms between the wildtype and the K294E mutant were kept in the construction of the principal components, but the procedure is otherwise identical.

### Projection grid and unbiased bin number

Each structure was projected onto the first two principal components. These points (PC1, PC2) were placed on an equally spaced 2-D grid. The number of bins along each axis was chosen *via* Sturges’ rule to avoid ad-hoc parameter tuning.

### Free-energy surface

For every bin i, we counted the occupancy P_i, converted it to a free energy *via*

$$\text{Δ}G_{i}=-k_{B}T\;\ln\left(\frac{P_{i}}{P_{\max}}\right)$$

Each free energy surface is zeroed to the global minima found.

### Identification of basins (minima) and assignment of frames

To locate true metastable wells on the noisy discrete surface, we applied an automatic minima finder (find_minima_find_structures.py, which can be found at https://github.com/salsburygroup/Salsbury_group_codes.). In brief, the script (i) Gaussian-smooths the ΔG matrix $\left(\text{σ}=0.5\;\text{bin}\right)$, (ii) uses an 8-connected minimum filter to flag local minima, (iii) labels each minimum and writes its grid coordinates, and (iv) maps every frame back to the nearest labelled minimum, returning both per-well populations and the frame IDs belonging to each basin Because the algorithm operates solely on the grid values, no clustering radius or user intervention is required, ensuring an unbiased delineation of conformational basins. The centroid of the lowest free energy bin within each well is then found and is considered the best structure representative of each minima.

A comprehensive discussion of our analysis methodology, except for the PCA analysis and its applications to biomolecular systems can be found in our earlier review (Godwin et al., 2015). All the codes used for all analyses, as well as the addition of missing atoms, can be found at https://github.com/salsburygroup/Salsbury_group_codes.

## Supplementary Material

Supp 1

Supplemental data for this article can be accessed online at https://doi.org/10.1080/07391102.2025.2563689.

## Figures and Tables

**Table 1.** Previously determined NSP2 structures.

References	Structure	Description	Tag	Open/closed CTR	Space Group	Symmetry	CTR status
Jayaram et al. (2002)	1L9V	SA11 NSP2	cHis	Closed	I422	D4	Structured
Taraporewala et al. (2006)	2GU0	Human NSP2 Bristol Group C	cHis	Closed	I4	D4	Structured
Kumar et al. (2007)	2R7C	SA11 NSP2 with nucleotides	cHis	Closed	I422	D4	Structured
Hu et al. (2012)	4G0A	SA11 NSP2 with RNA	No tag	Open/Closed	P3_1_21	D4	Structured
Criglar et al. (2018)	6CY9	SA11 NSP2 with disulfide bridge	No tag	Closed	I422	D4	Unstructured res. 296, 297
Criglar et al. (2018)	6AUK	SA11 NSP2 S313D	No tag	Closed	I422	D4	Structured
Criglar et al. (2018)	6CYA	SA11 NSP2 S313A	No tag	Closed	I422	D4	Unstructured res. 296–298
Bravo et al. (2021)	7PKO	SA11 Cryo-EM NSP2	cHis	Closed	N/A	D4	Structured
Nichols et al. (2024)	9OFT	SA11 NSP2	No tag	Closed	I422	D4	Structured
Nichols et al. (2024)	9OGQ	SA11 NSP2-K294E	No tag	Closed	I422	D4	Unstructured res. 296–298

**Table 2.** Data collection and refinement values.

Data collection and refinement	Untagged NSP2_WT_	Untagged NSP2_K294E_
Space group	I422	I422
Unit cell (Å)	a = b = 108.6, c = 153.4	a = b = 108.4, c = 153.9
Unit cell (°)	α = β = γ = 90°	α = β = γ = 90°
Molecules/asymmetric unit	1	1
Resolution (Å)	34.4–2.95 (3.04–2.95)	46.4–2.25 (2.32–2.25)
<I>/<sigI>	13.9 (4.7)	25.9 (1.6)
Average redundancy	12.1 (13.3)	13.1 (12.2)
Completeness (%)	100 (100)	100 (100)
CC1/2	99.7 (93.4)	99.8 (78.9)
R_factor_ (%)	22.8	22.80
R_free_ (%)	26.6	25.27
Wilson B_factor_	29.8	36.7
rmsd bond angles	0.822	0.663
rmsd bond lengths	0.005	0.005
Ramachandran plot (% favored, allowed, outliers)	95.2, 4.16, 0.64	96.7, 3.3, 0

Values for the highest resolution bins are shown in parentheses.

**Table 3.** RMSD overlay of molecular dynamics minima.

WT	K294E	RMSD (Å)	Area affected
9OFT	9OGQ	0.366	Overall similar
9OFT v. 1L9V		0.332	Overall similar
1L9V	9OGQ	0.392	Overall similar
Bin A	Bin F	2.663	C-term
Bin A	Bin G	1.944	C-term
Bin A	Bin H	2.058	Global
Bin A	Bin I	2.018	Similar, light N-term
Bin B	Bin F	2.334	Global
Bin B	Bin G	2.198	C-term
Bin B	Bin H	2.095	C-term
Bin B	Bin I	2.51	C-term
Bin C	Bin F	2.015	Light C-term
Bin C	Bin G	2.865	C-term
Bin C	Bin H	2.324	C-term
Bin C	Bin I	2.92	Global
Bin D	Bin F	2.105	Global
Bin D	Bin G	2.709	C-term
Bin D	Bin H	2.214	Global
Bin D	Bin I	2.816	C-term
Bin E	Bin F	2.292	C-term
Bin E	Bin G	3.017	C-term
Bin E	Bin H	2.547	C-term
Bin E	Bin I	3.148	C-term

The closer the value is to 0 means structures are more similar than different.

**Table 4.** Populations of NSP2 Conformational States.

NSP2_WT_ Bins	% Population	NSP2_K294E_ Bins	% Population
A	41.01	F	41.01
B	40.6	G	37.1
C	15.7	H	21.19
D	2.65	I	0.69
E	0.04		

## Data Availability

Protein structures were deposited into RCSB and PDB numbers have been indicated.
