# Supplementary figures and images for: K294E change in the rotavirus factory forming protein NSP2 stabilizes a rare C-terminal conformation
Source: J Biomol Struct Dyn. Author manuscript; Available in PMC 2025 Nov 19. (PMC12623717; doi:10.1080/07391102.2025.2563689)

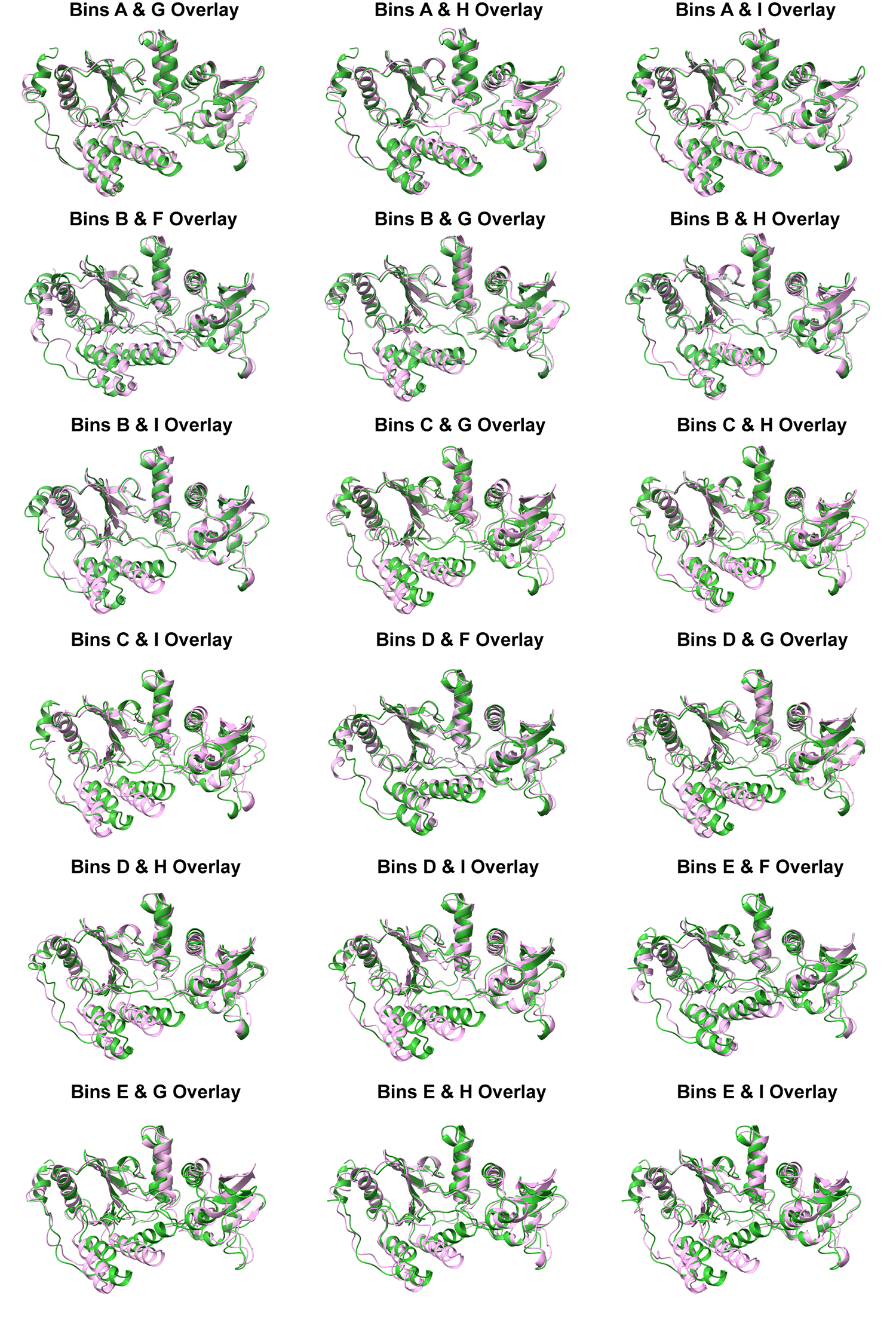

Supplement: Supp 1 [file NIHMS2114799-supplement-Supp_1.tif]
